# Distinct Bile Acid Signature in Parkinson's Disease With Mild Cognitive Impairment

**DOI:** 10.3389/fneur.2022.897867

**Published:** 2022-07-04

**Authors:** Kun Nie, Yanyi Li, Jiahui Zhang, Yuyuan Gao, Yihui Qiu, Rong Gan, Yuhu Zhang, Lijuan Wang

**Affiliations:** ^1^Department of Neurology, Guangdong Provincial People's Hospital, Guangdong Academy of Medical Sciences, Guangdong Neuroscience Institute, Guangzhou, China; ^2^Shantou University Medical College, Shantou, China

**Keywords:** Parkinson's disease, mild cognitive impairment, bile acid metabolites, chenodeoxycholic acid, ursodeoxycholic acid, cholic acid

## Abstract

**Backgrounds:**

Bile acid (BA) plays a crucial role in various neurodegenerative diseases, including Parkinson's disease (PD). However, no clinical evidence supports BA's potential role in patients with PD with mild cognitive impairment (PD-MCI).

**Objectives:**

This study aimed at investigating the differential BA profile between patients with PD-MCI and those with normal cognitive function (PD-NC).

**Methods:**

Ultra-high performance liquid chromatography-MS/MS was applied for BA quantitation. After between-group differences of the BA profile were addressed, orthogonal projections to latent structures—discriminant analysis (OPLS-DA) and the area under the receiver-operating-characteristic curve (AUC-ROC) were implemented for further verification.

**Results:**

Lower levels of chenodeoxycholic acid (CDCA), cholic acid (CA), and ursodeoxycholic acid (UDCA) were significantly associated with PD-MCI (*p* < 0.01 for both; VIP ≈ 2.67, 1.66, and 1.26, respectively). AUC-ROC were 78.1, 74.2, and 74.5% for CDCA, CA, and UDCA, respectively.

**Conclusion:**

CA, CDCA, and UDCA might be distinct BA signatures for patients with PD-MCI.

## Introduction

Cognitive impairment is considered one of the common non-motor symptoms (NMS) of Parkinson's disease (PD). There has been increasing awareness of the high incidence and damaging effects of cognitive impairment in PD in recent years. At the time when a PD diagnosis is established, almost all patients with PD suffer from some level of cognitive impairment (1). However, when the severity of cognitive impairment is not enough to significantly impair patients' daily life and functional independence, it is often ignored by patients or physicians in clinical practice (2). Although severe cognitive decline is not inevitable in all cases, patients with PD are six times more likely to develop dementia than the general population (3), significantly affecting patient functioning. Caring for someone with Parkinson's disease dementia (PDD) poses profound emotional, physical, and financial challenges for families and communities. Although there are some palliative treatments for the symptoms of dementia, there are no known disease-modifying treatments for PDD (1).

Mild cognitive impairment in PD (PD-MCI) is defined as an intermediate cognitive stage between PD with normal cognition (PD-NC) and PDD. Compared with patients with PD-NC, patients with PD-MCI have a higher risk of developing into PDD (4). Longitudinal studies have shown that the annual probability of patients with PD-MCI progressing to PDD is 6–15%, and the conversion rate of progressing to PDD within 5 years is about 39% (5). Within 3 years of follow-up, 28% of patients with PD-MCI returned to a state with normal cognitive function (6). When follow-up time was longer than 3 years, the reversion rate decreased, while the conversion rates to MCI and dementia increased (6). Therefore, PD-MCI is a critical period for early intervention of PDD. Accurate identification of PD-MCI and early intervention can help reduce the probability of PD-MCI converting to PDD.

The Movement Disorder Society (MDS) Task Force on MCI in PD has established an operative definition of PD-MCI to homogenize clinical practice and research (7). According to this definition, the formal diagnosis of PD-MCI requires the presence of four key features and the absence of exclusion criteria, mainly based on a comprehensive neuropsychological assessment. However, this has some limitations: first, it is time-consuming. In addition, the neuropsychological assessment results are subjective and affected by many factors, such as the patient's mental state and the severity of motor symptoms, so it is difficult to guarantee the homogeneity of results. Therefore, there is an urgent need to develop reliable diagnostic markers for PD-MCI, including new biochemical markers.

Bile acids (BA) are amphoteric molecules that mainly act as a solvent for dietary lipids and fat-soluble vitamins. The liver synthesizes primary bile acids, including cholic acid (CA), chenodeoxycholic acid (CDCA), and their conjugated form with glycine or taurine (8). Under the action of intestinal microorganisms, bile acids entering the intestine will undergo a multi-step metabolic reaction to generate secondary bile acids, such as deoxycholic acid (DCA), lithocholic acid (LCA), and ursodeoxycholic acid (UDCA) (9). Both primary and secondary BAs are present in the brains of mice with the ability to cross the blood-brain barrier (10–13). In recent years, BA signaling has been found to play a role in various neurodegenerative diseases (9, 14). Changes in BA metabolism and potential BA markers were observed in PD studies using rodent models (15–19). Li et al. found the upregulation of gut bacteria responsible for secondary bile acid synthesis and increased secondary bile acid DCA and LCA in patients with PD (20). Previous studies have also found that bile acids are associated with changes in cognitive impairment. Gut microbiota affect cognitive function by regulating primary bile acid metabolism in patients with Alzheimer's disease (AD) (21). However, bile acid has not been studied in PD cognitive impairment, and its specificity and sensitivity as biomarkers for early diagnosis are still unclear.

Plasma is an ideal sample for biomarker discovery and clinical diagnosis because it is simple to collect, relatively noninvasive, and easy to analyze. To test our hypothesis that the patients with PD-MCI showed changes in the bile acid profile, we performed a metabonomics analysis of plasma bile acids in the patients with PD-MCI and the patients with PD-NC.

## Materials and Methods

### Objects

This study was conducted in the Department of Neurology, Guangdong Provincial People's Hospital, Guangzhou, People's Republic of China. A diagnosis of idiopathic PD was assigned according to the MDS-Sponsored Revision of the Unified Parkinson's Disease Rating Scale (MDS-UPDRS) (7) by 2 neurologists with experience in movement disorders. Exclusion criteria included: (1) patients with secondary Parkinsonism or Parkinson-plus syndrome; (2) patients with hyperlipidemia or diabetes mellitus; (3) patients with a tumor, heart failure, chronic obstructive pulmonary disease, nephritis, infectious disease, or other severe chronic diseases; (4) a history of cerebrovascular disease, intracranial surgery, head trauma, AD, epilepsy, mental illness, or other conditions that may affect cognitive function; (5) patients failing to cooperate or complete the trial.

Demographic information was collected from each subject, including sex, gender, age, education level, age of the onset, duration, medications, smoking, and drinking history. A detailed medical history and physical examination were collected from all the participants. Unified Parkinson's Disease Rating Scale Part III (UPDRS III) and Hoehn-Yahr Scale (H-Y) were used to assess motor function. Mental status was assessed by Hamilton Anxiety Scale (HAMA) and Hamilton Depression Scale (HAMD).

Our neuropsychological protocol included Mini Mental State Examination (MMSE) and Montreal Cognitive Assessment (MoCA) to assess general cognitive functioning, memory impairment, and executive dysfunction. The attention/working memory domain was tested by Symbol Digit Modalities Test (SDMT) and Digit Span Test (DST). Executive functions were evaluated by the Verbal Fluency Test (VFT) and Clock Drawing Test. Memory was assessed by visual reproduction (VR) and logical memory. Language function was tested by the Similarities Test and Category Fluency Task. Visuo-spatial and visuo-perceptive functions were assessed by the Block design Test. The patients were diagnosed with PD-MCI based on Movement Disorders Society Task Force Level II 20 using a cut-off of 1.5 standard deviations below standard scores in one or more cognitive domains in neuropsychological tests as previously described (7).

### Serum Bile Acid Levels

A fasting venous blood sample was collected with a lithium heparin tube from all the subjects in the morning. The serum was separated and stored in the refrigerator at −80°C until experimental analysis. Thirty BA metabolites in serum were detected by ultra-high performance liquid chromatography-MS/MS (UHPLC-MS/MS). After quality-control checks, the data set included 19 BAs (11 were too few for statistical analysis) for a total of 67 subjects.

### Statistical Analysis

Statistical analyses were performed using SPSS25 software. Between-group (PD-MCI vs. PD-NC) differences in terms of demographics, clinical data, and BA characteristics were addressed by independent-sample *t*-tests or Mann–Whitney *U*-test for continuous variables and χ^2^ for categorical variables. False discovery rate (FDR)-based multiple comparison adjustment with the Benjamini-Hochberg procedure was used for BAs comparison to increase the significance of the findings.

To simplify the complexity of the high-dimensional metabolome data, orthogonal projections to latent structures—discriminant analysis (OPLS-DA) was implemented to identify the differential expression of patients with BAs in PD with or without cognitive impairment. Two iterations of the permutation test verified the model. Through OPLS-DA, a variable importance in projection (VIP) value could be obtained for each metabolite. The larger the VIP is, the more significant the contribution of the metabolite to distinguish the two groups. Differential metabolites were defined as VIP >1.0 and FDR-corrected *p*-values < 0.05. Then, according to the HMDB database, these differentially expressed metabolites were chemically classified. The area under the receiver-operating-characteristic curve (AUC-ROC) was also calculated.

### Ethical Approval

This study was performed following the principles outlined in the Declaration of Helsinki. Informed consent was obtained from all the individual participants included in the study.

## Results

### Demographic Characteristics

A total of 33 patients with PD-NC and 34 patients with PD-MCI were enrolled in this study. Demographic and clinical characteristics for all the subjects are shown in Table 1. Categorical data are displayed as absolute numbers and continuous data as mean ± standard error. The demographic characteristics of the two groups were compared, including gender, age, and education level, and the differences were not statistically significant (*p* > 0.05), indicating comparability (Table 1).

**Table 1:** Sample demographic and clinical characteristic.

**Demographics**	**PD-MCI**	**PD-NC**	***p*-value**
	**(*n* = 34)**	**(*n* = 33)**	
Gender (male/female), *n*	19/15	17/16	0.72
Age, years	62.9 ± 9.8	61.0 ± 10.3	0.44
Education, years	9.6 ± 3.7	10.5 ± 3.6	0.38
BMI, kg/m^2^	22.7 ± 2.8	23.7 ± 2.1	0.125
TC, mmol/L	4.8 ± 1.1	4.8 ± 1.0	0.851
HDL, mmol/L	1.3 ± 0.3	1.3 ± 0.3	0.692
LDL, mmol/L	3.1 ± 0.8	2.9 ± 0.8	0.913
TG, mmol/L	1.2 ± 0.7	1.2 ± 0.7	0.939
FBG, mmol/L	5.0 ± 1.1	4.8 ± 0.8	0.186
Disease duration, years	4.6 ± 0.7	3.5 ± 0.5	0.503
Age at onset, years	58.7 ± 1.6	57.8 ± 1.9	0.717
Hoehn and Yahr stage	2.1 ± 0.4	2.0 ± 0.5	0.31
UPDRS III, score	30.2 ± 15.4	25.9 ± 10.1	0.175
Family history (sporadic/familial), *n*	34/0	32/1	0.493
MoCA, score	20.0 ± 3.9	25.1 ± 2.5	<0.001
MMSE, score	26.2 ± 2.6	28.2 ± 1.7	0.007
HAMD, score	15.2 ± 8.1	10.7 ± 7.7	0.502
HAMA, score	12.8 ± 7.5	9.9 ± 6.7	0.872

*PD-MCI, Parkinson's disease with mild cognitive impairment; PD-NC, Parkinson's disease with normal cognition; BMI, body mass index; TC, total cholesterol; HDL, high-density lipoprotein cholesterol; LDL, low-density lipoprotein cholesterol; TG, triglyceride; FBG, fasting blood glucose*.

### Bile Acid Levels

**Figure 1:**
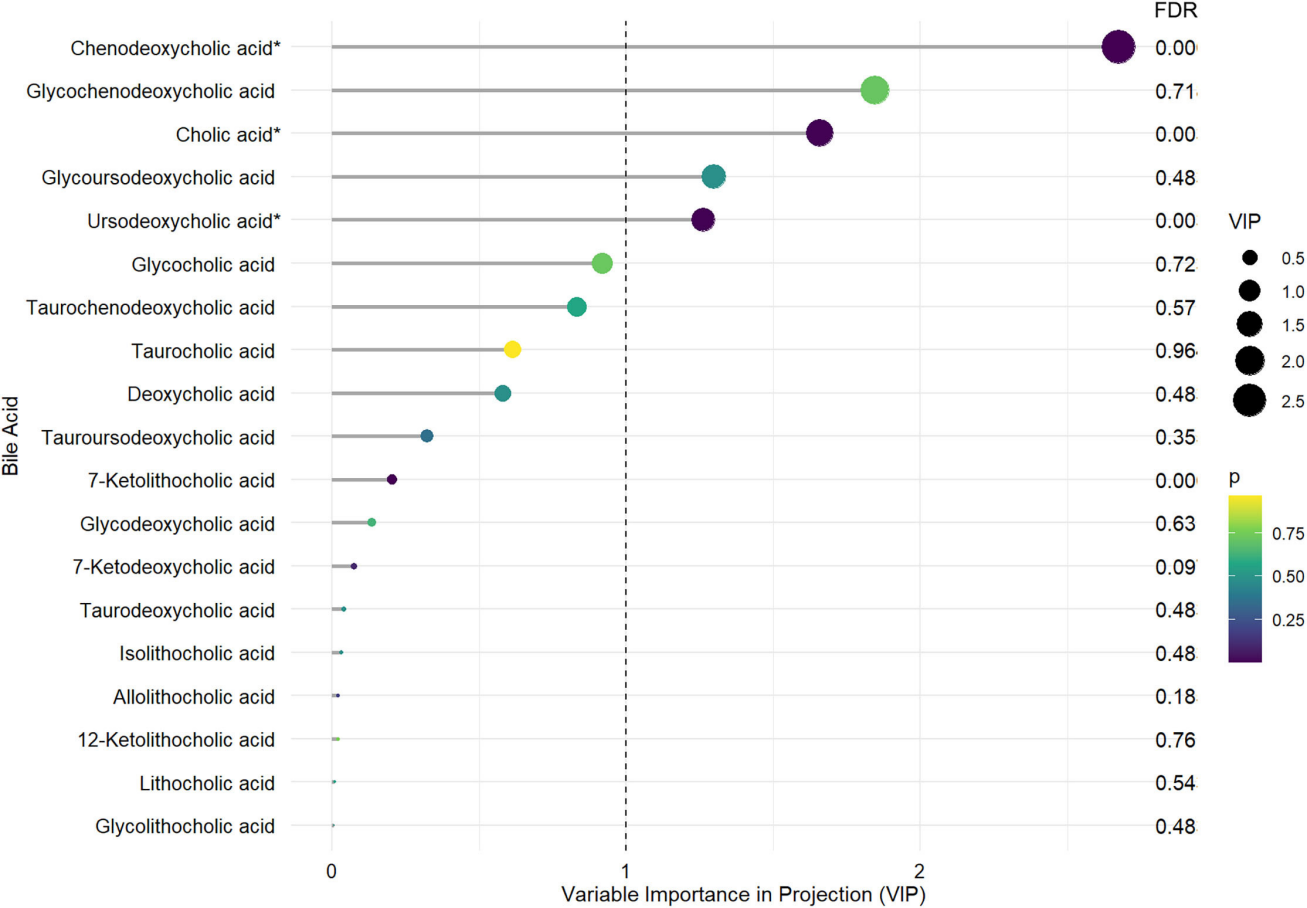
The distinct bile acid profile in PD-MCI compared to patients with PD-NC. The length of the lines and the size of the dots represent the value of variable importance in projection (VIP); the longer the lines and larger the dots, the larger the VIP value. The color of the dots represents the FDR-corrected *p*-value; the purple the color, the lower the *p*-value, and the yellower the color, the larger the *p*-value. ^*^Differential BA metabolites with VIP >1. and FDR-corrected *p*-values < 0.05.

**Figure 2:**
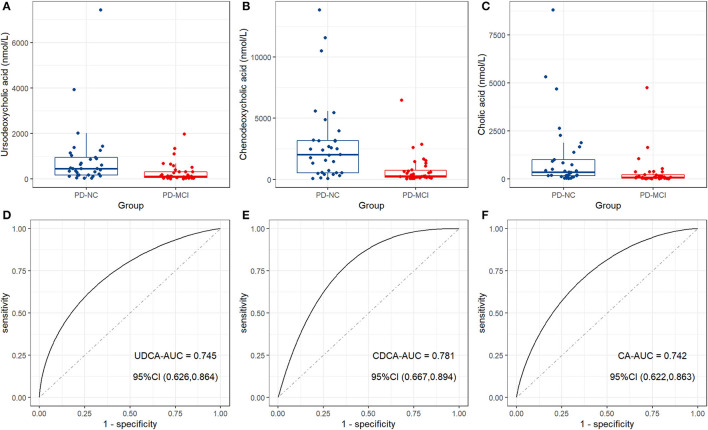
The distinct bile acid profile in patients with PD-MCI compared to patients with PD-NC. Box plots show the median (the horizontal line), 25–75 percentile (the box), and 5–95 percentile (the whisker) of the three BAs with VIP > 1 and FDR-corrected *p*-value < 0.05: **(A)** Ursodeoxycholic acid (UDCA); **(B)** chenodeoxycholic acid (CDCA); **(C)** cholic acid (CA). ROC curve analysis shows the diagnostic efficacy of CA **(D)**, CDCA **(E)**, and UDCA **(F)** for distinguishing between patients with PD-MCI and patients with PD-NC.

Mean and standard errors of 19 detected BAs are presented in Table 2. After controlling for multiple testing using FDR, lower levels of cholic acid (CA), chenodeoxycholic acid (CDCA), ursodeoxycholic acid (UDCA), and 7-Ketolithocholic acid were significantly associated with PD-MCI compared to patients with PD-NC (corrected *p* < 0.01). The OPLS-DA model was also constructed to show statistical differences in CDCA (VIP ≈ 2.67), Glycochenodeoxycholic acid (GCDCA, VIP ≈ 1.846), CA (VIP ≈ 1.658), glycoursodeoxycholic acid (GUDCA, VIP ≈ 1.298), and UDCA (VIP ≈ 1.26) levels between the PD-MCI and PD-NC groups. We identified CA, CDCA, and UDCA with VIP > 1 and corrected *p*-value < 0.05 as the differential BA metabolites in patients with PD-MCI (Figure 1; Table 2). The data distribution characteristics of these 3 BAs are shown in Figures 2A–C. AUC-ROC for CA, CDCA, and UDCA were 74.2, 78.1, and 74.5%, respectively (Figures 2D–F; Table 2).

**Table 2:** The bile acid profile in different groups of patients.

**Bile Acid**	**PD-MCI**	**PD-NC**	***p*-value**	**FDR**	**VIP**	**AUC-ROC**
**(nmol/L)**	**(*n* = 34)**	**(*n* = 33)**				
Allolithocholic acid	2.00 ± 5.10	0.83 ± 3.41	0.057	0.185	0.019	0.584
Isolithocholic acid	10.03 ± 15.46	11.38 ± 30.81	0.294	0.483	0.032	0.570
Lithocholic acid	9.22 ± 17.50	7.43 ± 18.43	0.367	0.543	0.01	0.553
7-Ketolithocholic acid	18.05 ± 39.57	52.95 ± 47.64	<0.001	<0.001	0.205	0.796
12-Ketolithocholic acid	5.78 ± 14.44	8.28 ± 23.00	0.714	0.76	0.018	0.521
Ursodeoxycholic acid*	**299.35** **±428.31**	**879.46** **±1,397.03**	**0.001**	**0.003**	**1.261**	**0.745**
Chenodeoxycholic acid*	**791.57** **±1,222.29**	**2,900.76** **±3,309.88**	**<0.001**	**<0.001**	**2.674**	**0.781**
Deoxycholic acid	524.52 ± 517.22	755.61 ± 783.28	0.264	0.483	0.579	0.579
7-Ketodeoxycholic acid	3.09 ± 8.42	12.99 ± 19.82	0.025	0.097	0.073	0.627
Cholic acid*	**334.84** **±845.71**	**1,104.95** **±1,871.77**	**0.001**	**0.003**	**1.658**	**0.742**
Glycolithocholic acid	21.22 ± 40.82	15.32 ± 43.59	0.296	0.483	0.006	0.558
Glycoursodeoxycholic acid	567.34 ± 566.87	1,279.30 ± 1,866.39	0.254	0.483	1.298	0.581
Glycochenodeoxycholic acid	2,617.73 ± 2,330.14	4,468.74 ± 6,505.48	0.598	0.718	1.846	0.537
Glycodeoxycholic acid	684.61 ± 761.80	771.66 ± 1,522.07	0.494	0.631	0.134	0.549
Glycocholic acid	759.72 ± 971.44	1,502.95 ± 3,443.99	0.643	0.723	0.918	0.533
Tauroursodeoxycholic acid	12.96 ± 30.19	58.95 ± 105.51	0.129	0.355	0.323	0.587
Taurochenodeoxycholic acid	310.07 ± 371.97	652.99 ± 1,214.39	0.415	0.571	0.831	0.588
Taurodeoxycholic acid	89.73 ± 107.45	115.28 ± 290.27	0.302	0.483	0.038	0.572
Taurocholic acid	116.21 ± 202.91	465.34 ± 1,365.00	0.959	0.964	0.614	0.504

*PD-MCI, Parkinson's disease with mild cognitive impairment; PD-NC, Parkinson's disease with normal cognition; FDR, false discovery rate*.

^*^Bile acid with VIP >1 and FDR-corrected p-values < 0.05.

*The bold values indicates the bile acids with VIP greater than 1.0 and FDR-corrected p values less than 0.05*.

### Specific Bile Acid and Different Cognitive Domains

After determining the differential bile acids between the PD-MCI and PD-NC groups, we conducted correlation analysis between these bile acid levels and the scores of various cognitive scales to find the specific cognitive domains affected by bile acids. Unfortunately, we found no significant correlation between bile acid levels and specific cognitive domains.

## Discussion

Currently, the mechanisms of cognitive impairment in Parkinson's disease are not fully understood. There is a lack of effective therapeutic strategies to alleviate or delay cognitive deterioration in Parkinson's disease (1). Evidence shows that PD-MCI is an independent risk factor in the occurrence of PDD (4) and is a critical period for early intervention of PDD. Accurate identification of PD-MCI and early intervention can help reduce the probability of cognitive deterioration to PDD. Identifying biomarkers for PD-MCI is an essential step toward improving current diagnostic criteria. In this study, we, for the first time, explored changes in bile acid profiles in patients with PD-MCI, which may provide clues to understand the disease mechanism of PD-MCI and be used to identify abnormal biochemical pathways and therapeutic targets for new effective drugs.

Metabolomics data are high dimensional and massive. There might be highly relevant characteristics between certain variables. Univariate analysis, such as *t*-test, cannot quickly, thoroughly, and accurately mine the potential information in the metabolomics data. To reduce the risk of false-positive errors and model overfitting, we combined univariate analysis and OLPS-DA, one of the multivariate analysis methods, to explore the distinct BA profile in the patients with PD-MCI. We found that CA, CDCA, and UDCA were significantly decreased in the patients with PD-MCI compared with those with normal cognition.

We also found that certain types of BAs were statistically significant only in univariate analysis or multivariate analysis. For instance, although the corrected *p*-value for 7-ketolithocholic acid was <0.001, the VIP value was small. On the one hand, that is probably because of the different analytic perspectives that the two types of statistical methods offer. Univariate analysis pays more attention to the independent change of the metabolite levels, while multivariate analysis concentrates more on the influence of the metabolites in the metabolic process. On the other hand, the negative results might be explained by the small statistical power related to the small sample size of this study. In other words, there are significant changes in these BAs, but the statistical methods showed false-negative results.

### The Changed Bile Acid Profile and Possible Mechanisms

The existence of BA is an emerging research topic in neurodegenerative disease (9). CA and CDCA are the only primary bile acids synthesized in humans, impacting fat digestion and energy metabolism signaling (22, 23). A previous study has shown that CBJC, a combination of Chinese herb active components, including cholic acid, can ameliorate the IBO-induced dementia in rats (24). CDCA has been shown to play a significant neuroprotective role in cerebrotendinous xanthomatosis (25, 26). Besides, CDCA significantly attenuates the cognitive dysfunction and spatial deficits induced by AlCl3 in the rat model (27). UDCA, another critical bile acid we discovered, is converted from CDCA catalyzed by 7α-hydroxysteroid dehydrogenase (7α-HSDH) and 7β-HSDH (28, 29). UDCA has been widely used in treating primary biliary cirrhosis and proved to have good safety and tolerability (30). It crosses the blood-brain barrier in a dose-dependent manner and improves mitochondrial function with anti-apoptotic effects in AD and PD models.

At the micro-level, BAs might be associated with cognitive functions in PD through the theory of neuroinflammation and apoptosis. CDCA could bind with Takeda G-protein-coupled receptor 5 (TGR5), a membrane receptor that alleviates neuroinflammation and inhibits cell apoptosis (31, 32). Besides, daily intraperitoneal injection of UDCA can significantly reduce the levels of nuclear factor-κB (NF-κB), Bcl2-related X apoptotic regulator (Bax), and caspase-9 mRNA, thus exerting anti-apoptotic effects (32). The interaction of BAs with anti-apoptotic substances may explain the relationships between BA and PD-MCI in some ways.

At a macro-level, abnormalities in BA metabolism may affect disease progression by altering structural networks in specific brain regions. Researchers have found that changes in BA of AD and patients with MCI were associated with changes in particular areas in brain imaging (21), which may also happen in patients with PD-MCI. We would combine metabolomics and radiomics to explore the association between BA, specifically CA, CDCA, and UDCA, and brain imaging changes in future studies.

### Bile Acids and Gut Microbiome

Bidirectional biochemical communication between the brain and the gut is associated with various neurodegenerative diseases, including Parkinson's disease. α-synuclein, a pathological marker of PD, was found to accumulate in the gastrointestinal tract of patients with early PD (33). After intestinal flora obtained from feces of patients with PD was transplanted into the intestinal tract of germ-free rats, all the previously disease-free rats developed PD symptoms (34). This suggests that the gut microbiome might be a vital contributor to Parkinson's disease. In terms of cognitive impairment, our previous studies have found that patients with PD-MCI have unique gut microbial characteristics, suggesting that intestinal flora may be related to the pathogenic mechanism of PD-MCI.

Intestinal flora is an integral part of bile acid metabolism. In rats with PD symptoms that overexpress α- synuclein, intestinal microbial diversity was changed, and the levels of related primary bile acids were significantly increased and thus affecting motor symptoms (16). Another study has found that intestinal bacteria adjust the primary bile acid metabolism in patients with AD and, in turn, affect cognitive function (21). Increased levels of metabolites of intestinal microbiota, including deoxycholic acid and glycocholic acid, were significantly associated with thinning of cortical regions in bilateral frontal, parietal, and temporal lobes (21). This suggests that bile acids may serve as a “bridge” between gut microbiome disorder and the occurrence and development of cognitive impairment in PD.

### Limitations

Because the inclusion criteria were relatively strict, the total number of samples included was relatively small, which might decrease statistical power and cause some false-negative results. In addition, only the patients with PD-MCI and the patients with PD-NC were included in this study to explore the bile acid metabolites in PD cognitive impairment preliminarily. We will recruit more patients with PD with cognitive impairment in future studies, including those with dementia.

## Data Availability Statement

The raw data supporting the conclusions of this article will be made available by the authors, without undue reservation.

## Ethics Statement

The studies involving human participants were reviewed and approved by Research Ethics Committee, Guangdong Provincial People's Hospital, Guangdong Academy of Medical Sciences. The patients/participants provided their written informed consent to participate in this study.

## Author Contributions

KN, YL, and JZ contributed to conception and design of the study. KN and JZ organized the database. JZ performed the statistical analysis. YL wrote the first draft of the manuscript. KN, JZ, YQ, RG, and YG wrote sections of the manuscript. All the authors contributed to manuscript revision and read and approved the submitted version.

## Funding

This work was supported by Science and Technology Planning Project of Guangzhou (202102080054); Guangdong Medical Research Foundation (A2021308); Science and Technology Planning Project of Guangdong Province (2020A0505140006).

## Conflict of Interest

The authors declare that the research was conducted in the absence of any commercial or financial relationships that could be construed as a potential conflict of interest.

## Publisher's Note

All claims expressed in this article are solely those of the authors and do not necessarily represent those of their affiliated organizations, or those of the publisher, the editors and the reviewers. Any product that may be evaluated in this article, or claim that may be made by its manufacturer, is not guaranteed or endorsed by the publisher.
